# Magnetic Mesoporous Carbon/β-Cyclodextrin–Chitosan Nanocomposite for Extraction and Preconcentration of Multi-Class Emerging Contaminant Residues in Environmental Samples

**DOI:** 10.3390/nano11020540

**Published:** 2021-02-20

**Authors:** Geaneth Pertunia Mashile, Anele Mpupa, Philiswa Nosizo Nomngongo

**Affiliations:** 1 Department of Chemical Sciences, University of Johannesburg, Doornfontein Campus, P.O. Box 17011, Doornfontein 2028, South Africa; petmashile2009@hotmail.com (G.P.M.); anelempupa@yahoo.com (A.M.); 2 Department of Science and Innovation (DSI)/National Research Foundation (NRF) South African Research Chair (SARChI): Nanotechnology for Water, University of Johannesburg, Doornfontein 2028, South Africa; 3 Department of Science and Innovation (DSI)/Mintek Nanotechnology Innovation Centre, University of Johannesburg, Doornfontein 2028, South Africa

**Keywords:** anticonvulsants and β-blockers, parabens, mesoporous carbon, fluoroquinolone, β-cyclodextrin, global concentration

## Abstract

This study reports the development of magnetic solid-phase extraction combined with high-performance liquid chromatography for the determination of ten trace amounts of emerging contaminants (fluoroquinolone antibiotics, parabens, anticonvulsants and β-blockers) in water systems. Magnetic mesoporous carbon/β-cyclodextrin–chitosan (MMPC/Cyc-Chit) was used as an adsorbent in dispersive magnetic solid-phase extraction (DMSPE). The magnetic solid-phase extraction method was optimized using central composite design. Under the optimum conditions, the limits of detection (LODs) ranged from 0.1 to 0.7 ng L^−1^, 0.5 to 1.1 ng L^−1^ and 0.2 to 0.8 ng L^−1^ for anticonvulsants and β-blockers, fluoroquinolone and parabens, respectively. Relatively good dynamic linear ranges were obtained for all the investigated analytes. The repeatability (*n* = 7) and reproducibility (*n* = 5) were less than 5%, while the enrichment factors ranged between 90 and 150. The feasibility of the method in real samples was assessed by analysis of river water, tap water and wastewater samples. The recoveries for the investigated analytes in the real samples ranged from 93.5 to 98.8%, with %RSDs under 4%.

## 1. Introduction

The use of chemicals such as preservatives, pharmaceuticals, plasticizers, perfumes, UV filters and microplastics, among others, is universal [1]. Among these chemicals, pharmaceutical and personal care products (PPCPs) have attracted a lot of interest due to their extensive use in the prevention or treatment of human and animal diseases as we are improving the quality of life [2]. Evidence indicates that conventional water treatment processes for the removal of PPCPs such as coagulation, filtration, disinfection and flocculation, amongst others, are ineffective in the complete removal of PPCPs. These chemicals have been detected in different environmental compartments at concentration levels ranging from ng L^−1^ to μg L^−1^ [3,4,5]. In addition, the detection of PPCPs in South African surface waters has been poorly evaluated and has only increased in recent years [6,7,8,9,10,11,12,13,14,15]. The presence of PPCPs in the environment is of global concern, as they are said to be biologically active [2]. As a results of their biological activity and endocrine disruptive effects, they can pose serious health effects to living organisms and the environment [2]. Therefore, it is important to investigate the occurrence of PPCPs in different environmental compartments.

Thus far, several analytical techniques, such as capillary electrophoresis (CE) [2,16,17], high-performance liquid chromatography (HPLC) [18,19,20], liquid chromatography–mass spectrometry (LC-MS) [21], ultra-performance liquid chromatography coupled with tandem mass spectrometry [1,2,3] and gas chromatography–mass spectrometry (GC-MS) [22,23,24,25], have been employed for quantification of PPCPs in numerous matrices. Owing to matrix effects of complex environmental samples and trace levels of PPCPs, sample cleanup prior to analytical detection and quantification is required. For this reason, different preconcentration and extraction procedures such as liquid-phase microextraction techniques (LPME) [26], traditional solid-phase extraction (SPE) [2,16], dispersive solid-phase extraction (DSPE) [19,27,28], solid-phase microextraction (SPME) [29], stir bar sorptive extraction (SBSE) [21,30] and supramolecular solvent-based LPME [20,21,30,31,32], among others, have been developed for PPCPs analysis.

Among the abovementioned techniques, adsorbent-based extraction methods have received significant attention in recent years. This is due to the use of different solid-phase materials that can be tuned based on target analytes. Until now, different kinds of adsorbents have been used for extraction and preconcentration of PPCPs. These include multi-walled carbon nanotubes [33], nanofibers [27,28,34], graphene oxide nanocomposites [19,35], biopolymer-based composites [36,37], activated carbon, metal–organic frameworks [2,38] and metal oxide nanocomposites [28]. Recently, our previous research prepared a biodegradable superabsorbent based on a magnetic mesoporous carbon/β-cyclodextrin–chitosan (MMPC/CycChit) nanocomposite for removal of fluoroquinolones (FQs) from environmental samples [39].

In addition to sample pretreatment, complete chromatographic separation is necessary for the selective and sensitive detection of target analytes, especially with a UV detector [40]. The separation of pharmaceuticals in HPLC-DAD is mostly achieved using stationary phases containing n-octylsilyl- (C8) and n-octadecylsilyl- (C18) functional groups bound to the silica surface through reverse-phase liquid chromatographic (RPLC) separation [41]. However, the choice of a suitable mobile phase could allow the achievement of good separation. Acetonitrile and methanol are widely used organic mobile phases for the HLPC separation of pharmaceuticals [40,41]. Researchers have reported that in order to achieve a better resolution, shorter retention times and reproducible results, a mixture of acetonitrile and methanol together with the use of additives (such as formic acid, acetate buffer and phosphoric acid) can be used [42,43].

The objective of this work was to develop a rapid, robust and simple method for extraction and preconcentration of anticonvulsants, beta-blockers, parabens and fluoroquinolones (FQs) in environmental samples. The method was based on dispersive magnetic solid-phase extraction (DMSPE) based on the previously reported MMPC/CycChit adsorbent combined with high-performance liquid chromatography with diode array detection (HPLC-DAD). The choice of analytes to be investigated was based on the previous studies which revealed that anticonvulsants, beta-blockers, parabens and fluoroquinolones (FQs) are frequently detected in South African water systems [7,31,36,44,45]. The experimental factors (mass of adsorbent, eluent type, eluent volume, extraction time, desorption time and sample pH) affecting the extraction and preconcentration procedure were optimized using univariate and multivariate approaches. According to our knowledge, no studies have been performed on the analysis of emerging multi-class pollutants using dispersive magnetic solid-phase extraction with an MMPC/CycChit nanocomposite as the adsorbent.

## 2. Materials and Methods

### 2.1. Materials

Ethanol, methanol (MeOH) (HPLC grade), acetonitrile (ACN) (HPLC grade) and ortho-phosphoric acid were acquired from Sigma-Aldrich (St. Louis, MO, USA). Standards of the target analytes were obtained from Sigma and their corresponding information is illustrated in Table 1. The stock solution containing fluoroquinolones (FQs), parabens, β-blockers and anticonvulsants was prepared by dissolving appropriate amounts of analytes of interest in methanol. These analytes included danofloxacin (DANO), enrofloxacin (ENRO), levofloxacin (LEVO), atenolol (ANL), propranolol hydrochloride (PPNL), carbamazepine (CBZ), methylparaben (MP), ethylparaben (EP), propylparaben (PP) and butylparaben (BP). The simulated sample solution was prepared by diluting appropriate volumes of stock solution with tap water free from the target analytes. A set of calibration standards was prepared from stock solution and diluted with ultrapure water. The stock solution was stored in the refrigerator at 4–8 °C, and the simulated sample solutions were prepared daily. The simulated solutions were used throughout the method development stages. Details of chromatographic conditions and other instrumentations used are presented in Section S1 in the Supplementary Information.

### 2.2. Samples and Sample Collection

Both wastewater samples, raw (influent) and treated (effluent), used in this study were collected at different points in the Daspoort Wastewater Treatment Plant (Pretoria, Gauteng, South Africa), while river and tap water samples were collected from the Apies River and the laboratory. The samples were collected using pre-cleaned 500 mL glass bottles. After sampling, the water samples were stored at 4 °C for a maximum of 1 week until being analyzed.

### 2.3. UA-MSPDE Preconcentration Procedure

The extraction procedure was conducted based on a literature report by [9]. Briefly, 20–60 mg of an adsorbent (MMPC/Cyc-Chit) was added in sample glass bottles. Simulated sample solution (20 mL) at a concentration level of 100 µg L^−1^ for each analyte was placed in the sample bottles containing respective masses of MMPC/Cyc-Chit adsorbent. The extraction and preconcentration steps were assisted by ultrasonication for 10–30 min. The analyte-loaded adsorbent was separated from the sample via an external magnet, and the liquid phase was discarded. Subsequently, the analytes were eluted from the adsorbent using 2 mL of eluent solvent. The capabilities of different eluent solvents were in investigated. These include acetonitrile (ACN), ultrapure deionized water, methanol (MeOH), mixture of ACN/MeOH (50:50), mixture of ACN/H_2_O (50:50), mixture of MeOH/H_2_O (50:50) and 0.01 mol L^−1^ H_3_PO_4_/CAN. The elution process was achieved by ultrasonic dispersion for 10 min. Similarly, the eluent solvent was separated from the adsorbent by magnetic decantation, and the analytes in the eluent solvent were analyzed using HPLC-DAD. The effect of independent variables, that is, extraction time (10–30 min), mass of adsorbent (20–60 mg) and sample pH (4–9), were evaluated using central composite design (CCD) at 5 levels. The percentage recoveries of each investigated analyte were used as the dependent variable (analytical response). Nineteen randomized experiments were performed, eight at the factorial points, six at the axial points and five at the central point.

### 2.4. Quality Assurance/Quality Control (QA/QC)

The quality assurance/quality control (QA/QC) of the DMSPE-HPLC-DAD method was performed according to our previous study [46]. Firstly, blank samples were injected to the HPLC system and none of the target analytes were detected. These results provided assurance that blank correction from all investigated samples was not necessary. During the analysis of the samples, standard solutions of each analyte at 10 and 100 ng L^−1^ were used as QA/QC samples. Blank samples processed in a similar manner to real samples and above-mentioned QA/QC standard solutions were analyzed after every tenth sample. However, when samples were less than ten, the QA/QC procedure was followed after every three samples.

## 3. Results and Discussion

### 3.1. Optimization of Desorption Conditions

**Figure 1:**
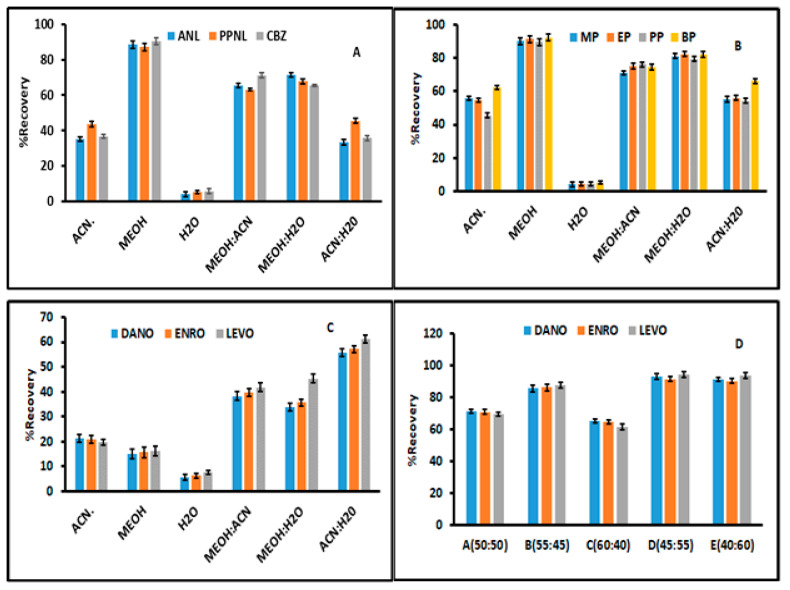
Recoveries of different elution volumes using different solvents for residual analytes. (**A**) Anticonvulsants and β-blockers (atenolol (ANL), propranolol hydrochloride (PPNL) and carbamazepine (CBZ)), (**B**) parabens (methylparaben (MP), ethylparaben (EP), propylparaben (PP) and butylparaben (BP)), (**C**,**D**) fluoroquinolones (danofloxacin (DANO), enrofloxacin (ENRO), levofloxacin (LEVO)).

The elution process of the adsorbates from the adsorbent was investigated in order to attain the highest percentage recoveries of the analytes. The selection of a suitable eluent is important because of the differences in physicochemical properties of organic solvents used as eluents and the analytes to be desorbed. In this study, acetonitrile (ACN), ultrapure deionized water, methanol (MeOH), a mixture of ACN/MeOH (50:50), a mixture of ACN/H_2_O (50:50) and a mixture of MeOH/H_2_O (50:50) were used for the elution of propranolol, atenolol, carbamazepine, fluoroquinolones and parabens. The desorption process was carried out via ultrasonication, and preliminary experiments showed that five minutes was long enough to attain quantitative recoveries. As seen, methanol was found to the best solvent (Figure 1A,B) for the desorption of β-blockers, CBZ and parabens. This suggested that β-blockers, CBZ and parabens were highly soluble in methanol. However, all the investigated desorption solvents were not suitable for elution of fluoroquinolones (Figure 1C). This might be because of the strong π–π or electrostatic interactions between the FQs and the nanocomposite [47,48,49]. As seen in Figure 1C, the mixture of acetonitrile and water had recoveries greater than 50%. Therefore, the desorption capabilities of different mixtures of 0.01 mol L^−1^ H_3_PO_4_ and ACN were investigated. The result obtained revealed that the quantitative recoveries were obtained with when 55:45 (*v/v*) of the 0.01 mol L^−1^ H_3_PO_4_/ACN mixture was used (Figure 1D). This suggested that the acidified desorption solution led to the cationic forms of FQs. Moreover, the surface of the adsorbent at lower pH values is positive. This phenomenon resulted in electrostatic repulsion between the analytes and the adsorbent, thus promoting the desorption of FQs from the surface of the nanocomposite. For further studies, methanol and mixtures of 0.01 mol L^−1^ H_3_PO_4_/ACN (45:55 *v/v*) were selected as suitable eluents. These findings are in line with previous studies [31].

### 3.2. Optimization of the Preconcentration Procedure

**Figure 2:**
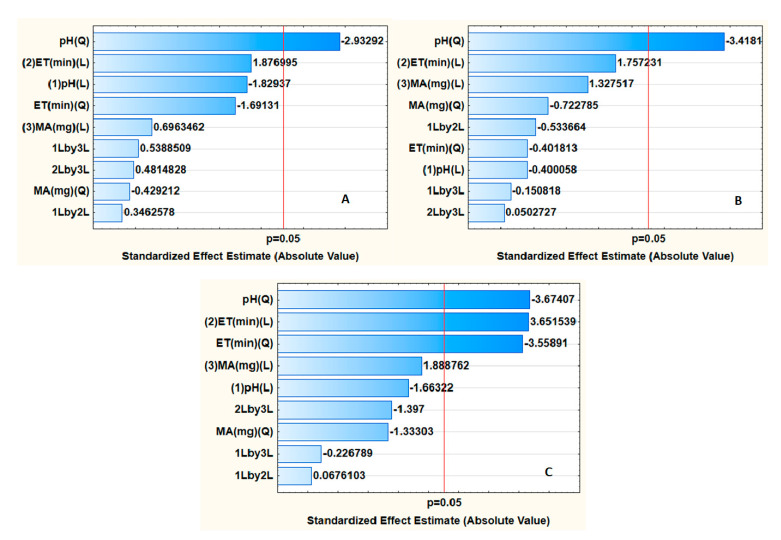
Pareto chart for standardized effects on variables for preconcentration of (**A**) β-blockers and anticonvulsants, (**B**) fluoroquinolones and (**C**) parabens.

To obtain the most satisfactory extraction and preconcentration conditions, the effect of various parameters (sample pH, mass of adsorbent and extraction time) was investigated. The optimization of these parameters was achieved using central composite design (CCD), and the design matrix together with the respective responses is reported in Supplementary Tables S1–S3. The experimental data were analyzed using analysis of variance (ANOVA) (Figure 2). Pareto charts for each analyte revealed that the sample pH and mass of adsorbent were significant at the 95% confidence level for all the analytes. In contrast, Figure 2C shows that the sample pH and mass of adsorbent and their interactions were statistically significant for the preconcentration of parabens.

The interactive effects of the investigated variables were examined using 3D response surface plots (Figures S1–S3). As observed from the ANOVA results, the sample pH proved to be one of the critical factors on the extraction and preconcentration of parabens. This was due to the fact that pH is known to have the ability to affect the charges of both the adsorbent and the analytes depending on the analyte pKa and pH at the point of zero charge (pH_PZC_) of the adsorbent [50]. When the sample pH is higher than the analyte pKa, the analyte remains in its neutral form. In contrast, pH ≤ pKa results in the protonation of the analyte, which influences the adsorbent–analyte interaction. In the case of fluoroquinolones, the analytical response increased with increasing sample pH, and the %R was attained between pH 6 and 8. This is because fluoroquinolones can exist in three forms in aqueous systems, that is, cationic (pH > pKa_2_), zwitterionic (pKa_1_ ≤ pH ≤ pKa_2_) and anionic (pH < pKa_1_), and these forms are pH-dependent [39]. Consequently, the adsorption mechanism is also dependent on the adsorbent surface charge. Moreover, the extraction analytes depend on the adsorbent surface charge, and the pH_PZC_ of the nanocomposite used in this study was 8.0, implying that it bears a negative surface charge at pH values higher than 8 [39]. As seen in Figures S1–S3, percentage recoveries increased up to pH 8; after that, a significant decrease was observed. This might be due to the electrostatic repulsion between the analytes and adsorbent [33].

**Figure 3:**
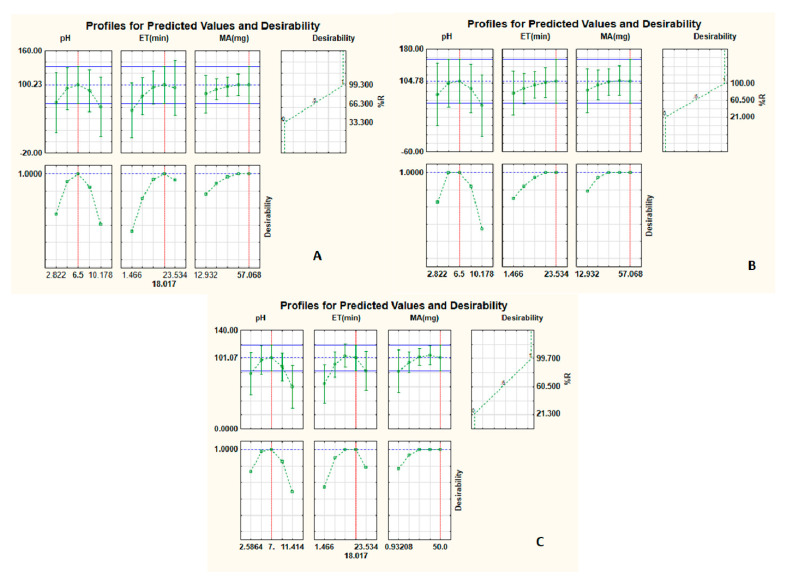
Desirability function for optimization of independent variables for (**A**) β-blockers and anticonvulsants, (**B**) fluoroquinolones and (**C**) parabens.

Figure 3 presents the desirability profiles and summary of the optimum conditions desired to obtain maximum recoveries of (A) β-blockers and anticonvulsants, (B) fluoroquinolones and (C) parabens. Figure 3 presents the individual desirability scores for the preconcentration of target analytes (left-hand side, bottom). The %R obtained from the plots for each parameter in the model is presented at the top left-hand side. According to Mashile et al. [36], the plots on the top left-hand side present the changes in the level of each individual variable and its analytical response as well as its overall desirability. According to Figure 3, the minimum, central and maximum %R values were 21–33.3%, 60.5–66.3% and 99.3–100%, respectively. These %R values correspond to desirability values of 0.0, 0.5 and 1.0. To obtain maximum recoveries of the target analytes, the desirability score of 1.0 was chosen as the target value for the optimization of the individual variables [36]. As seen from Figure 3A–C, the desirable recoveries were obtained at pH 6.5, ET 23 min and MA 57 mg for β-blockers, anticonvulsants and fluoroquinolones (Figure 3A,B), while a desirable recovery was obtained at pH 7, ET 23 min and MA 50 mg for parabens (Figure 3C). To validate the optimum conditions, preconcentration of target analytes was carried out using the optimal conditions, and the %R values ranged from 97.9 ± 2.1 to 98.7 ± 2.5%. The experimental values were in agreement with the predicted data obtained from the desirability function profile. Suggesting that a response surface methodology model based on central composite design was valid and appropriate for optimization of the DMSPE method.

### 3.3. Validation of the Preconcentration Method

The analytical performance of the DMSPE/HPLC-DAD method was assessed using limits of detection (LODs), limits of quantification (LOQs), the dynamic linear range, precision (repeatability and reproducibility), the enhancement factor and spike recovery tests. The LODs and LOQs were calculated from seven measurements of the lowest standard of the calibration at a signal-to-noise ratio (S/N) of 3 and 10. The linearity of the method was investigated using a series of standard solutions containing a mixture of target analytes at a concentration range of 0–500, 0–1500 and 0–300 µg L^−1^ for β-blockers and anticonvulsants, fluoroquinolones and parabens, respectively. Wide linearity with correlation coefficients (R^2^) up to 0.9993 was obtained. The repeatability (expressed as relative standard deviation (%RSD) was investigated by same-day analysis of 10 consecutive replicates of 100 ng L^−1^, while reproducibility (interday) %RSD experiments were conducted over a 5-day period. The analytical figures of merit results are summarized in Table 2 (detailed individual results are presented in Supplementary Data, Tables S4–S6). The analytical performance of the developed method was compared with other sorbent-based sample preparation techniques reported in the literature [1,22,23,51,52,53] (Table 3).

The applicability of the proposed method was assessed by analyzing β-blockers and anticonvulsants, fluoroquinolones and parabens in wastewater, river water and tap water samples. The samples were spiked at two levels with 50 and 100 ng L^−1^ for β-blockers, anticonvulsants and parabens and 5 and 20 ng L^−1^ for fluoroquinolones. The results obtained were used to evaluate the accuracy of the method. The spike recovery experiments were carried out in triplicates and the %RSD was estimated (Table 4, detailed individual results and typical chromatograms are presented in Supplementary Data, Tables S7–S9 and Figures S3–S6).

Pharmaceutical and personal care products have been identified and detected in almost all ecological compartments across the world. As seen, carbamazepine, levofloxacin and butylparaben were not detected in wastewater, river water and tap water samples (Tables S7–S9, Supplementary Data). Among the detected PPCPs, parabens were found at the highest concentration in influent wastewater, i.e., 937 ± 10 ng L^−1^ for methylparaben and 781 ± 11 ng L^−1^ for propylparaben (Table S9). In addition, most studied analytes were not detected in tap water samples, except methylparaben (3.89 ± 0.09 ng L^−1^) and propylparaben (4.81 ± 0.05 ng L^−1^). The concentrations of anticonvulsants, beta-blockers, parabens and fluoroquinolones were compared with those reported in other countries. As seen, the levels for beta-blockers, anticonvulsants and fluoroquinolones obtained in this study were within the lower end of the ranges reported in the literature (Table 5). Paraben levels were lower than those reported in some parts of South Africa (up to 1988 ng L^−1^), Egypt (up to 6380 ng L^−1^), Kenya (30–1160 ng L^−1^), China (up to 5960 ng L^−1^), Turkey (17,000–33,000 ng L^−1^) and the United Kingdom (2642–11,601 ng L^−1^). Furthermore, paraben concentrations were found to be higher than those reported in Pakistan, Portugal, Spain, Brazil and Poland (Table 5).

## 4. Conclusions

This study described the broad use of a sample pretreatment procedure with the application of a versatile biodegradable supersorbent MMPC/Cyt-Chit for the extraction and preconcentration of multi-class PPCPs from environmental water samples, where preconcentration of multi-class PPCPs was carried out by the simple dispersive magnetic solid-phase extraction technique prior to chromatographic detection. Evaluations for the suitable elution solvent and sorbent pH were performed in order to select the best extraction conditions. Based on the results obtained, methanol was the best desorption solvent, while the zeta potential of the nanocomposite indicated that it was positively charged at pH below 8 and negatively charged at pH above 8.0, making it a suitable material for the preconcentration of analytes with a high or low pKa value. Moreover, the combination of a nanocomposite with properties such as a large specific surface area and predominantly porous structure with the DMSPE technique yielded high recoveries up to 99% for all analytes. The DMSPE-HPLC-DAD technique was also tested in spiked water samples where it demonstrated that the linearity of the method ranged from 0.05 to 400, 0.05 to 300, 0.10 to 350 and 0.2 to 1000 µg L^−1^ for beta-blockers, parabens, anticonvulsants and fluoroquinolones, respectively. In addition, their LODs ranged from 0.0045 to 0.07 µg L^−1^ with a correlation coefficient (R^2^) of up to 0.9991 for all PPCPs analyzed. Therefore, this indicated that the optimized DMSPE-HPLC-DAD technique was suitable for the simultaneous preconcentration of multi-class PPCPs from different aquatic matrices. The method also proved to be sensitive and cost-effective as less time and sorbent mass were used to simultaneously extract and quantify all analytes before HPLC determination.

## Figures and Tables

**Table 1:** List of pharmaceutical and personal care product (PPCP) compounds, chemical structures, molecular masses and pK_a_ values.

Analytes	Class	Chemical Structures	Molecular Mass (g mol^−1^)	pKa Values
Danofloxacin	Fluoroquinolones	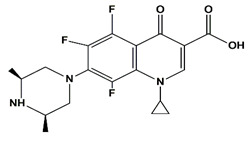	357.37	6.22 and 9.43
Enrofloxacin	Fluoroquinolones	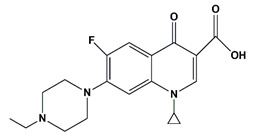	359.4	6.19 and 7.59
Levofloxacin	Fluoroquinolones	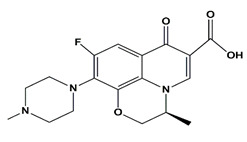	361.368	6.02 and 8.15
Atenolol	β-blockers	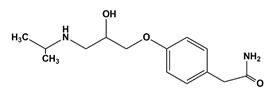	266.336	9.6
Propranolol hydrochloride	β-blockers	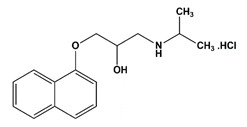	295.80	9.4
Carbamazepine	Anticonvulsant	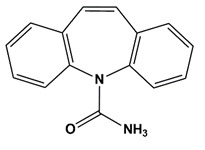	236.27	13.9
Methyl paraben	Preservatives	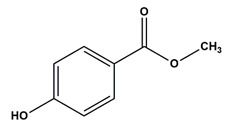	152.15	8.3
Ethyl paraben	Preservatives	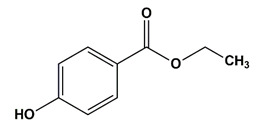	166.17	8.3
Propyl paraben	Preservatives	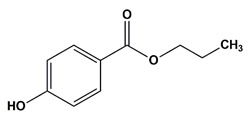	180.20	8.2
Butyl paraben	Preservatives	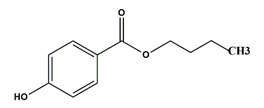	194.23	8.2

**Table 2:** Summary of analytical characteristics of the DMSPE-HPLC-DAD method for determination of β-blockers and anticonvulsants, fluoroquinolones and parabens.

Analytical Performances	β-Blockers and Anticonvulsants	Fluoroquinolones	Parabens
LODs (ng L^−1^)	0.1–0.7	0.45–1.1	0.2–0.8
LOQs (ng L^−1^)	0.33–2.3	1.5–3.7	0.67–2.7
Linearity (µg L^−1^)	LOQ-400	LOQ-1000	LOQ-300
R^2^	0.9987–0.9991	0.9979–0.9990	0.9987–0.9993
Repeatability (%RSD)	1.9–2.5	1.8–3.4	1.5–2.7
Reproducibility (%RSD)	3.1–4.3	2.8–4.4	2.9–4.4

**Table 3:** Comparison of the present study with other solid-phase extraction methods for analysis of multi-class pollutants.

Adsorbent/Method	Mass of Adsorbent (mg)	Analytes	LOD (µg L^−1^)	Linearity (µg L^−1^)	Correlation Coefficient (R^2^)	Refs.
MM-CMC/IT-SPME-HPLC-FLD	N/A	DANO, ENRO	0.14–0.61	0.001–5.0	0.9980	[51]
Oasis HLB-SPE-LC/MS/MS	60	Atenolol, carbamazepine	1.01–69.30	1.87–138.6	0.9669–0.9999	[52]
Carbowax 20M/FPSE/GC-MS	Not indicated	EP, BP	0.009–0.021	0,05–500	0.9992–0.9997	[22]
OasisHLB/ RDSE/GC-MS	40	MP, EP, PP, BP	0.02–0.15	0.06–0.44	0.9904–0.9989	[23]
Mixed mode cationic exchange cartidges (MXC)	60	MP	0.01	0.06–1122	0.9999	[1]
C18,Floracil, QuEChERS/UPLC-QqQ-MS	50 mg	**Beta-blockers:** Atenolol, propranolol; **Preservatives**: BP, MP, PP; **Anticonvulsant**: carbamazepine	0.093–0.12	1.0–200.0	>0.95	[53]
MMPC/Cyc-Chit/HPLC-DAD	50 mg	**Beta-blockers:** atenolol, propranolol	0.0001–0.0007	LOQ-400	0.9987–0.9991	**This work**
		**Parabens**: MP, EP, PP, BP	0.0001–0.0007	LOQ-300	0.9987–0.9993	
		**Anticonvulsant:** carbamazepine	0.0003	LOQ-350	0.9989	
		**Quinolones**: DANO, LEVO, ENRO	0.00045–0.0011	LOQ-1000	0.9987–0.9990	

**Table 4:** Determination of β-blockers and anticonvulsants, fluoroquinolones and parabens in real water samples (*n* = 3).

Samples	Analytes	Initial (ng L^−1^)	Found (ng L^−1^) ^a^	%R	%RSD	Found (ng L^−1^) ^b^	%R	%RSD
Influent	β-blockers, anticonvulsants	ND-28.9	42.7–72.1	86.3–94.4	2.8–4.5	93.3–118	87.8–93.3	1.6–3.4
	Fluoroquinolones	ND-7.33	4.87–12.1	93.8–97.3	3.9–4.2	19.1–33.9	92.8–100	3.7–4.3
	Parabens	1.37–937	46.2–984	89.7–94.3	2.3–3.1	92.7–1078	91.3–96.1	2.6–3.5
Effluent	Β-blockers, anticonvulsants	ND-7.65	48.4–55.4	95.4–98.7	3.1–4.3	95.5–105	96.8–97.1	1.7–4.9
	Fluoroquinolones	ND-2.07	4.92–6.88	96.2–98.3	2.4–3.2	19.3–21.3	94.4–98.1	2.1–3.1
	Parabens	ND-43.1	48.7–92.7	97.3–99.1	1.9–2.4	96.5–141	96.5–97.9	1.8–2.8
Tap water	β-blockers, anticonvulsants	ND	48.9–49.6	97.7–99.1	2.6–3.5	98.9–99.5	98.0–99.5	2.0–4.0
	Fluoroquinolones	ND	4.91–4.98	98.1–99.6	1.4–1.8	19.8–19.9	98.9–99.5	1.7–1.8
	Parabens	ND-4.81	49.0–53.8	97.9–99.3	1.9–2.4	97.8–104	97.8–99.3	1.3–1.6
River water	β-blockers, anticonvulsants	ND-4.92	49.5–53.3	97.0–98.9	2.2–4.2	99.1–102	97.0–99.1	1.7–2.9
	Fluoroquinolones	ND-3.12	4.94–8.02	94.8–98.7	1.9–2.5	19.4–22.4	95.6–96.3	1.0–1.4
	Parabens	ND-40.1	48.5–88.8	96.6–98.8	1.7–2.4	97.1–137	96.6–98.2	1.3–1.9

^a^ Samples spiked with 5 ng L^−1^ for fluoroquinolones and 50 ng L^−1^ for β-blockers, anticonvulsants and parabens. ^b^ Samples spiked with 20 ng L^−1^ for fluoroquinolones and 100 ng L^−1^ for β-blockers, anticonvulsants and parabens.

**Table 5:** Global concentrations (ng L^−1^) of β-blockers and anticonvulsants, fluoroquinolones and parabens in environmental samples.

Country	β-Blockers	Anticonvulsants	Fluoroquinolones	Parabens	References
South Africa	0.96–39,000	4.0–94	110–2257	0–1988	[31,36,44,45,54]
Latvia	0–150	18–50	250–400	-	[55]
Egypt	0–187	0–342	-	0–6380	[56,57,58]
Kenya	-	0–430	-	30–1160	[59,60]
Spain	10–6066	28–283	0–2153	14–720	[21,61,62,63]
Italy	0–57	0–137	-	-	[64]
Pakistan	0.99–452	11–15	2–37,000	110–228	[65,66]
China	0–995	23–115	0–2032	0–5960	[65,66,67,68,69,70,71]
Brazil	0.02–1.89	69		90–788	[72,73,74,75]
Canada	114	20	34	-	[76,77,78]
Poland	69–205	2.0	248.7	0.01–5.03	[79,80,81,82]
Portugal	220–690	0.32–1.60		2.1–51	[83,84]
Turkey		0.92–24.25		17,000–33,000	[85]
United Kingdom	93–388	13–56	180	2642–11,601	[86,87]
South Africa	0–28	-	0–43	0–937	This study

## Supplementary Materials

The following are available online at https://www.mdpi.com/2079-4991/11/2/540/s1, Table S1: CCD matrix and analytical response: preconcentration of beta-blockers and CBZ, Table S2: CCD matrix and analytical response: preconcentration of fluroquinolones, Table S3: CCD matrix and analytical response: preconcentration of parabens, Table S4: Analytical characteristics of the DMSPE-HPLC-DAD method for determination of atenolol, propranolol and carbamazepine, Table S5: Analytical characteristics of the DMSPE-HPLC-DAD method for determination of DANO, ENRO and LEVO, Table S6: Analytical characteristics of the DMSPE-HPLC-DAD method for determination of four parabens, Table S7: Determination of atenolol, propranolol and carbamazepine in real water samples (ng L−1, n = 3), Table S8: Determination of DANO, ENRO and LEVO in real water samples (ng L−1, n = 3), Table S9: Determination of MP, EP, PP and BP in real water samples (ng L^−1^, *n* = 3), Figure S1: 3D plots for the interaction of optimum parameters for preconcentration of beta-blockers and anticonvulsants, Figure S2: 3D plots for the interaction of optimum parameters for preconcentration of three fluoroquinolones, Figure S3: 3D plots for the interaction of optimum parameters for preconcentration of four parabens, Figure S4: Typical chromatograms for preconcentration of beta-blockers and anticonvulsants in influent samples, Figure S5: Typical chromatograms for preconcentration of fluoroquinolones in effluent samples, Figure S6: Typical chromatograms for preconcentration of parabens in influent samples.

## References

[1] Proctor K., Petrie B., Barden R., Arnot T., Kasprzyk-Hordern B. (2019). Multi-residue ultra-performance liquid chromatography coupled with tandem mass spectrometry method for comprehensive multi-class anthropogenic compounds of emerging concern analysis in a catchment-based exposure-driven study. Anal. Bioanal. Chem..

[2] Li Q., Li Q., Guo S., Li D., Wo R., Zhao R., Jiang W. (2019). Composite Material that Comprised Metal–Organic Nanotubes and a Sponge as a High-Performance Adsorbent for the Extraction of Pharmaceuticals and Personal Care Products from Environmental Water Samples. Chem. Asian J..

[3] Petrie B., Youdan J., Barden R., Kasprzyk-Hordern B. (2016). Multi-residue analysis of 90 emerging contaminants in liquid and solid environmental matrices by ultra-high-performance liquid chromatography tandem mass spectrometry. J. Chromatogr. A.

[4] Papageorgiou M., Kosma C., Lambropoulou D. (2016). Seasonal occurrence, removal, mass loading and environmental risk assessment of 55 pharmaceuticals and personal care products in a municipal wastewater treatment plant in Central Greece. Sci. Total Environ..

[5] Parpounas A., Litskas V., Hapeshi E., Michael C., Fatta-Kassinos D. (2017). Assessing the presence of enrofloxacin and ciprofloxacin in piggery wastewater and their adsorption behaviour onto solid materials, with a newly developed chromatographic method. Environ. Sci. Pollut. Res..

[6] Wood T.P., Duvenage C.S.J., Rohwer E. (2015). The occurrence of anti-retroviral compounds used for HIV treatment in South African surface water. Environ. Pollut..

[7] Rimayi C., Chimuka L., Gravell A., Fones G.R., Mills G.A. (2019). Use of the Chemcatcher® passive sampler and time-of-flight mass spectrometry to screen for emerging pollutants in rivers in Gauteng Province of South Africa. Environ. Monit. Assess..

[8] Ojemaye C.Y., Petrik L. (2019). Occurrences, levels and risk assessment studies of emerging pollutants (pharmaceuticals, perfluoroalkyl and endocrine disrupting compounds) in fish samples from Kalk Bay harbour, South Africa. Environ. Pollut..

[9] Mpupa A., Mashile G.P., Nomngongo P.N. (2019). Ultrasound-assisted dispersive solid phase nanoextraction of selected personal care products in wastewater followed by their determination using high performance liquid chromatography-diode array detector. J. Hazard. Mater..

[10] Madikizela L.M., Mdluli P.S., Chimuka L. (2016). Experimental and theoretical study of molecular interactions between 2-vinyl pyridine and acidic pharmaceuticals used as multi-template molecules in molecularly imprinted polymer. React. Funct. Polym..

[11] Madikizela L.M., Chimuka L. (2017). Simultaneous determination of naproxen, ibuprofen and diclofenac in wastewater using solid-phase extraction with high performance liquid chromatography. Water Sa.

[12] Madikizela L.M., Muthwa S.F., Chimuka L. (2014). Determination of triclosan and ketoprofen in river water and wastewater by solid phase extraction and high performance liquid chromatography. S. Afr. J. Chem..

[13] Amdany R., Moya A., Cukrowska E., Chimuka L. (2015). Optimization of the temperature for the extraction of pharmaceuticals from wastewater by a hollow fiber silicone membrane. Anal. Lett..

[14] Amdany R., Chimuka L., Cukrowska E., Kohoutek J., Vrana B. (2014). Investigating the temporal trends in PAH, PCB and OCP concentrations in Hartbeespoort Dam, South Africa, using semipermeable membrane devices (SPMDs). Water.

[15] Amdany R., Chimuka L., Cukrowska E. (2014). Determination of naproxen, ibuprofen and triclosan in wastewater using the polar organic chemical integrative sampler (POCIS): A laboratory calibration and field application. Water.

[16] Medrano L.C., Flores-Aguilar J.F., Islas G., Rodríguez J.A., Ibarra I.S. (2019). Solid-Phase Extraction and Large-Volume Sample Stacking-Capillary Electrophoresis for Determination of Artificial Sweeteners in Water Samples. Food Anal. Methods.

[17] Valimaña-Traverso J., Amariei G., Boltes K., García M.Á., Marina M.L. (2019). Enantiomer stability and combined toxicity of duloxetine and econazole on Daphnia magna using real concentrations determined by capillary electrophoresis. Sci. Total Environ..

[18] Morés L., da Silva A.C., Merib J., Dias A.N., Carasek E. (2019). A natural and renewable biosorbent phase as a low-cost approach in disposable pipette extraction technique for the determination of emerging contaminants in lake water samples. J. Sep. Sci..

[19] Rashvand M., Vosough M. (2016). Graphene oxide–polyaniline nanocomposite as a potential sorbent for dispersive solid-phase extraction and determination of selected pharmaceutical and personal care products in wastewater samples using HPLC with a diode-array detector. Anal. Methods.

[20] Chen J., Deng W., Li X., Wang X., Xiao Y. (2019). Hexafluoroisopropanol/Brij-35 based supramolecular solvent for liquid-phase microextraction of parabens in different matrix samples. J. Chromatogr. A.

[21] Mijangos L., Ziarrusta H., Olivares M., Zuloaga O., Möder M., Etxebarria N., Prieto A. (2018). Simultaneous determination of 41 multiclass organic pollutants in environmental waters by means of polyethersulfone microextraction followed by liquid chromatography–tandem mass spectrometry. Anal. Bioanal. Chem..

[22] Kaur R., Kaur R., Grover A., Rani S., Malik A.K., Kabir A., Furton K.G. (2019). Fabric phase sorptive extraction/GC-MS method for rapid determination of broad polarity spectrum multi-class emerging pollutants in various aqueous samples. J. Sep. Sci..

[23] Arismendi D., Becerra-Herrera M., Cerrato I., Richter P. (2019). Simultaneous determination of multiresidue and multiclass emerging contaminants in waters by rotating-disk sorptive extraction–derivatization-gas chromatography/mass spectrometry. Talanta.

[24] Kotowska U., Kapelewska J., Kotowski A., Pietuszewska E. (2019). Rapid and sensitive analysis of hormones and other emerging contaminants in groundwater using ultrasound-assisted emulsification microextraction with solidification of floating organic droplet followed by GC-MS Detection. Water.

[25] Lee S., Kim K., Jeon J., Moon H.-B. (2019). Optimization of suspect and non-target analytical methods using GC/TOF for prioritization of emerging contaminants in the Arctic environment. Ecotoxicol. Environ. Saf..

[26] Khan W.A., Arain M.B., Yamini Y., Shah N., Kazi T.G., Pedersen-Bjergaard S., Tajik M. (2019). Hollow fiber-based liquid phase microextraction followed by analytical instrumental techniques for quantitative analysis of heavy metal ions and pharmaceuticals. J. Pharm. Anal..

[27] Dimpe K.M., Nomngongo P.N. (2019). Application of activated carbon-decorated polyacrylonitrile nanofibers as an adsorbent in dispersive solid-phase extraction of fluoroquinolones from wastewater. J. Pharm. Anal..

[28] Lekota M.W., Dimpe K.M., Nomngongo P.N. (2019). MgO-ZnO/carbon nanofiber nanocomposite as an adsorbent for ultrasound-assisted dispersive solid-phase microextraction of carbamazepine from wastewater prior to high-performance liquid chromatographic detection. J. Anal. Sci. Technol..

[29] Portillo-Castillo O.J., Castro-Ríos R., Chávez-Montes A., González-Horta A., Cavazos-Rocha N., de Torres N.H.W., Garza-Tapia M. (2018). Developments of solid-phase microextraction fiber coatings for environmental pharmaceutical and personal care products analysis. Rev. Anal. Chem..

[30] Wang Z., He M., Chen B., Hu B. (2019). Azo-linked porous organic polymers/polydimethylsiloxane coated stir bar for extraction of benzotriazole UltraViolet absorbers from environmental water and soil samples followed by high performance liquid chromatography-diode array detection. J. Chromatogr. A.

[31] Selahle S.K., Nomngongo P.N. (2019). Supramolecular Solvent Based Liquid-Liquid Microextraction for Preconcentration of Selected Fluoroquinolone Antibiotics in Environmental Water Sample Prior to High Performance Liquid Chromatographic Determination. Curr. Anal. Chem..

[32] Gissawong N., Boonchiangma S., Mukdasai S., Srijaranai S. (2019). Vesicular supramolecular solvent-based microextraction followed by high performance liquid chromatographic analysis of tetracyclines. Talanta.

[33] Jakubus A., Gromelski M., Jagiello K., Puzyn T., Stepnowski P., Paszkiewicz M. (2019). Dispersive solid-phase extraction using multi-walled carbon nanotubes combined with liquid chromatography–mass spectrometry for the analysis of β-blockers: Experimental and theoretical studies. Microchem. J..

[34] Zhao R., Ma T., Li S., Tian Y., Zhu G. (2019). Porous Aromatic Framework Modified Electrospun Fiber Membrane as a Highly Efficient and Reusable Adsorbent for Pharmaceuticals and Personal Care Products Removal. Acs Appl. Mater. Interfaces.

[35] Delhiraja K., Vellingiri K., Boukhvalov D.W., Philip L. (2019). Development of Highly Water Stable Graphene Oxide-Based Composites for the Removal of Pharmaceuticals and Personal Care Products. Ind. Eng. Chem. Res..

[36] Mashile G., Mpupa A., Nomngongo P. (2018). In-syringe micro solid-phase extraction method for the separation and preconcentration of parabens in environmental water samples. Molecules.

[37] Zhuo N., Lan Y., Yang W., Yang Z., Li X., Zhou X., Liu Y., Shen J., Zhang X. (2017). Adsorption of three selected pharmaceuticals and personal care products (PPCPs) onto MIL-101 (Cr)/natural polymer composite beads. Sep. Purif. Technol..

[38] Seo P.W., Bhadra B.N., Ahmed I., Khan N.A., Jhung S.H. (2016). Adsorptive removal of pharmaceuticals and personal care products from water with functionalized metal-organic frameworks: Remarkable adsorbents with hydrogen-bonding abilities. Sci. Rep..

[39] Mashile G.P., Dimpe K.M., Nomngongo P.N. (2020). A Biodegradable Magnetic Nanocomposite as a Superabsorbent for the Simultaneous Removal of Selected Fluoroquinolones from Environmental Water Matrices: Isotherm, Kinetics, Thermodynamic Studies and Cost Analysis. Polymers..

[40] Pascale R., Bianco G., Coviello D., Cristina Lafiosca M., Masi S., Mancini I.M., Bufo S.A., Scrano L., Caniani D. (2020). Validation of a liquid chromatography coupled with tandem mass spectrometry method for the determination of drugs in wastewater using a three-phase solvent system. J. Sep. Sci..

[41] Petrović M., Hernando M.D., Díaz-Cruz M.S., Barceló D. (2005). Liquid chromatography–tandem mass spectrometry for the analysis of pharmaceutical residues in environmental samples: A review. J. Chromatogr. A.

[42] Miao X.-S., Koenig B.G., Metcalfe C.D. (2002). Analysis of acidic drugs in the effluents of sewage treatment plants using liquid chromatography–electrospray ionization tandem mass spectrometry. J. Chromatogr. A.

[43] Sacher F., Lange F.T., Brauch H.-J., Blankenhorn I. (2001). Pharmaceuticals in groundwaters: Analytical methods and results of a monitoring program in Baden-Württemberg, Germany. J. Chromatogr. A.

[44] Selahle S.K., Nomngongo P.N. (2020). Determination of fluoroquinolones in the environmental samples using vortex assisted dispersive liquid-liquid microextraction coupled with high performance liquid chromatography. Int. J.Environ. Anal. Chem..

[45] Rimayi C., Odusanya D., Weiss J.M., de Boer J., Chimuka L. (2018). Contaminants of emerging concern in the Hartbeespoort Dam catchment and the uMngeni River estuary 2016 pollution incident, South Africa. Sci. Total Environ..

[46] Lekota M.W., Mpupa A., Dimpe K.M., Nomngongo P.N. (2020). Preparation of ferric oxide-aluminium oxide carbon nanofiber nanocomposites for ultrasound-assisted dispersive magnetic solid phase extraction of 17-beta estradiol in wastewater. Emerg. Contam..

[47] Jiang W., Cui W.-R., Liang R.-P., Qiu J.-D. (2020). Zwitterionic surface charge regulation in ionic covalent organic nanosheets: Synergistic adsorption of fluoroquinolone antibiotics. Chem. Eng. J..

[48] Li Z., Xu F., Liu Z., Qin C., Ren H., Li Y. (2018). Facile synthesis of novel porous self-assembling hydrogen-bonding covalent organic polymers and their applications towards fluoroquinolone antibiotics adsorption. RSC Adv..

[49] Wen A., Li G., Wu D., Yu Y., Yang Y., Hu N., Wang H., Chen J., Wu Y. (2020). Sulphonate functionalized covalent organic framework-based magnetic sorbent for effective solid phase extraction and determination of fluoroquinolones. J. Chromatogr. A.

[50] Manouchehri M., Seidi S., Rouhollahi A., Noormohammadi H., Shanehsaz M. (2020). Micro solid phase extraction of parabens from breast milk samples using Mg-Al layered double hydroxide functionalized partially reduced graphene oxide nanocomposite. Food Chem..

[51] Pang J., Liao Y., Huang X., Ye Z., Yuan D. (2019). Metal-organic framework-monolith composite-based in-tube solid phase microextraction on-line coupled to high-performance liquid chromatography-fluorescence detection for the highly sensitive monitoring of fluoroquinolones in water and food samples. Talanta.

[52] Miossec C., Lanceleur L., Monperrus M. (2019). Multi-residue analysis of 44 pharmaceutical compounds in environmental water samples by solid-phase extraction coupled to liquid chromatography-tandem mass spectrometry. J. Sep. Sci..

[53] Rashid A., Wang Y., Li Y., Yu C., Sun Q. (2019). Simultaneous analysis of multiclass contaminants of emerging concern in sediments by liquid chromatography with tandem quadrupole mass spectrometry. Environ. Toxicol. Chem..

[54] Offiong N.-A.O., Inam E.J., Edet J.B. (2019). Preliminary Review of Sources, Fate, Analytical Challenges and Regulatory Status of Emerging Organic Contaminants in Aquatic Environments in Selected African Countries. Chem. Afr..

[55] Pugajeva I., Rusko J., Perkons I., Lundanes E., Bartkevics V. (2017). Determination of pharmaceutical residues in wastewater using high performance liquid chromatography coupled to quadrupole-Orbitrap mass spectrometry. J. Pharm. Biomed. Anal..

[56] Radwan E.K., Ibrahim M.B.M., Adel A., Farouk M. (2020). The occurrence and risk assessment of phenolic endocrine-disrupting chemicals in Egypt’s drinking and source water. Environ. Sci. Pollut. Res..

[57] Younes H.A., Mahmoud H.M., Abdelrahman M.M., Nassar H.F. (2019). Seasonal occurrence, removal efficiency and associated ecological risk assessment of three antibiotics in a municipal wastewater treatment plant in Egypt. Environ. Nanotechnol. Monit. Manag..

[58] Abdallah M.A.-E., Nguyen K.-H., Ebele A.J., Atia N.N., Ali H.R.H., Harrad S. (2019). A single run, rapid polarity switching method for determination of 30 pharmaceuticals and personal care products in waste water using Q-Exactive Orbitrap high resolution accurate mass spectrometry. J. Chromatogr. A.

[59] K’Oreje K.O., Kandie F.J., Vergeynst L., Abira M.A., Van Langenhove H., Okoth M., Demeestere K. (2018). Occurrence, fate and removal of pharmaceuticals, personal care products and pesticides in wastewater stabilization ponds and receiving rivers in the Nzoia Basin, Kenya. Sci. Total Environ..

[60] K’oreje K.O., Vergeynst L., Ombaka D., De Wispelaere P., Okoth M., Van Langenhove H., Demeestere K. (2016). Occurrence patterns of pharmaceutical residues in wastewater, surface water and groundwater of Nairobi and Kisumu city, Kenya. Chemosphere.

[61] Valcárcel Y., Valdehíta A., Becerra E., López de Alda M., Gil A., Gorga M., Petrovic M., Barceló D., Navas J.M. (2018). Determining the presence of chemicals with suspected endocrine activity in drinking water from the Madrid region (Spain) and assessment of their estrogenic, androgenic and thyroidal activities. Chemosphere.

[62] Čelić M., Gros M., Farré M., Barceló D., Petrović M. (2019). Pharmaceuticals as chemical markers of wastewater contamination in the vulnerable area of the Ebro Delta (Spain). Sci. Total Environ..

[63] Ziarrusta H., Val N., Dominguez H., Mijangos L., Prieto A., Usobiaga A., Etxebarria N., Zuloaga O., Olivares M. (2017). Determination of fluoroquinolones in fish tissues, biological fluids, and environmental waters by liquid chromatography tandem mass spectrometry. Anal. Bioanal. Chem..

[64] Mandaric L., Diamantini E., Stella E., Cano-Paoli K., Valle-Sistac J., Molins-Delgado D., Bellin A., Chiogna G., Majone B., Diaz-Cruz M.S. (2017). Contamination sources and distribution patterns of pharmaceuticals and personal care products in Alpine rivers strongly affected by tourism. Sci. Total Environ..

[65] Ashfaq M., Li Y., Rehman M.S.U., Zubair M., Mustafa G., Nazar M.F., Yu C.-P., Sun Q. (2019). Occurrence, spatial variation and risk assessment of pharmaceuticals and personal care products in urban wastewater, canal surface water, and their sediments: A case study of Lahore, Pakistan. Sci. Total Environ..

[66] Riaz L., Mahmood T., Kamal A., Shafqat M., Rashid A. (2017). Industrial release of fluoroquinolones (FQs) in the waste water bodies with their associated ecological risk in Pakistan. Environ. Toxicol. Pharm..

[67] Zhang Y., Duan L., Wang B., Liu C.S., Jia Y., Zhai N., Blaney L., Yu G. (2020). Efficient multiresidue determination method for 168 pharmaceuticals and metabolites: Optimization and application to raw wastewater, wastewater effluent, and surface water in Beijing, China. Environ. Pollut..

[68] Zhang X., Zhao H., Du J., Qu Y., Shen C., Tan F., Chen J., Quan X. (2017). Occurrence, removal, and risk assessment of antibiotics in 12 wastewater treatment plants from Dalian, China. Environ. Sci. Pollut. Res..

[69] Lu J., Mao H., Li H., Wang Q., Yang Z. (2017). Occurrence of and human exposure to parabens, benzophenones, benzotriazoles, triclosan and triclocarban in outdoor swimming pool water in Changsha, China. Sci. Total Environ..

[70] Ben W., Zhu B., Yuan X., Zhang Y., Yang M., Qiang Z. (2018). Occurrence, removal and risk of organic micropollutants in wastewater treatment plants across China: Comparison of wastewater treatment processes. Water Res..

[71] Liao C., Shi J., Wang X., Zhu Q., Kannan K. (2019). Occurrence and distribution of parabens and bisphenols in sediment from northern Chinese coastal areas. Environ. Pollut..

[72] Marta-Sanchez A.V., Caldas S.S., Schneider A., Cardoso S.M.V.S., Primel E.G. (2018). Trace analysis of parabens preservatives in drinking water treatment sludge, treated, and mineral water samples. Environ. Sci. Pollut. Res..

[73] Reichert G., Mizukawa A., Antonelli J., de Goulart F.A.B., Filippe T.C., de Azevedo J.C.R. (2020). Determination of Parabens, Triclosan, and Lipid Regulators in a Subtropical Urban River: Effects of Urban Occupation. Water Air Soil Pollut..

[74] Chaves M.d.J.S., Barbosa S.C., de Melo Mallinowski M., Volpato D., Castro Í.B., dos Santos Franco T.C.R., Primel E.G. (2020). Pharmaceuticals and personal care products in a Brazilian wetland of international importance: Occurrence and environmental risk assessment. Sci. Total Environ..

[75] Reis-Santos P., Pais M., Duarte B., Caçador I., Freitas A., Pouca A.S.V., Barbosa J., Leston S., Rosa J., Ramos F. (2018). Screening of human and veterinary pharmaceuticals in estuarine waters: A baseline assessment for the Tejo estuary. Mar. Pollut. Bull..

[76] Comtois-Marotte S., Chappuis T., Duy S.V., Gilbert N., Lajeunesse A., Taktek S., Desrosiers M., Veilleux É., Sauvé S. (2017). Analysis of emerging contaminants in water and solid samples using high resolution mass spectrometry with a Q Exactive orbital ion trap and estrogenic activity with YES-assay. Chemosphere.

[77] Krogh J., Lyons S., Lowe C.J. (2017). Pharmaceuticals and personal care products in municipal wastewater and the marine receiving environment near Victoria Canada. Front. Mar. Sci..

[78] Nakata H., Kannan K., Jones P.D., Giesy J.P. (2005). Determination of fluoroquinolone antibiotics in wastewater effluents by liquid chromatography–mass spectrometry and fluorescence detection. Chemosphere.

[79] Giebułtowicz J., Stankiewicz A., Wroczyński P., Nałęcz-Jawecki G. (2016). Occurrence of cardiovascular drugs in the sewage-impacted Vistula River and in tap water in the Warsaw region (Poland). Environ. Sci. Pollut. Res..

[80] Kot-Wasik A., Jakimska A., Śliwka-Kaszyńska M. (2016). Occurrence and seasonal variations of 25 pharmaceutical residues in wastewater and drinking water treatment plants. Environ. Monit. Assess..

[81] Kapelewska J., Kotowska U., Karpińska J., Kowalczuk D., Arciszewska A., Świrydo A. (2018). Occurrence, removal, mass loading and environmental risk assessment of emerging organic contaminants in leachates, groundwaters and wastewaters. Microchem. J..

[82] Wagil M., Kumirska J., Stolte S., Puckowski A., Maszkowska J., Stepnowski P., Białk-Bielińska A. (2014). Development of sensitive and reliable LC-MS/MS methods for the determination of three fluoroquinolones in water and fish tissue samples and preliminary environmental risk assessment of their presence in two rivers in northern Poland. Sci. Total Environ..

[83] de Jesus Gaffney V., Cardoso V.V., Cardoso E., Teixeira A.P., Martins J., Benoliel M.J., Almeida C.M.M. (2017). Occurrence and behaviour of pharmaceutical compounds in a Portuguese wastewater treatment plant: Removal efficiency through conventional treatment processes. Environ. Sci. Pollut. Res..

[84] Jonkers N., Sousa A., Galante-Oliveira S., Barroso C.M., Kohler H.-P.E., Giger W. (2010). Occurrence and sources of selected phenolic endocrine disruptors in Ria de Aveiro, Portugal. Environ. Sci. Pollut. Res..

[85] Guzel E.Y., Cevik F., Daglioglu N. (2019). Determination of pharmaceutical active compounds in Ceyhan River, Turkey: Seasonal, spatial variations and environmental risk assessment. Hum. Ecol. Risk Assess. Int. J..

[86] Gardner M., Jones V., Comber S., Scrimshaw M.D., Coello-Garcia T., Cartmell E., Lester J., Ellor B. (2013). Performance of UK wastewater treatment works with respect to trace contaminants. Sci. Total Environ..

[87] Kasprzyk-Hordern B., Dinsdale R.M., Guwy A.J. (2009). The removal of pharmaceuticals, personal care products, endocrine disruptors and illicit drugs during wastewater treatment and its impact on the quality of receiving waters. Water Res..

