# Supplementary material for: Magnetic Mesoporous Carbon/β-Cyclodextrin–Chitosan Nanocomposite for Extraction and Preconcentration of Multi-Class Emerging Contaminant Residues in Environmental Samples
Source: Nanomaterials (Basel). 2021 Feb 20;11(2):540. doi: 10.3390/nano11020540 (PMC7924173; doi:10.3390/nano11020540)
Supplement: Supplementary file 1 [file nanomaterials-11-00540-s001.pdf]

Magnetic Mesoporous Carbon/β-Cyclodextrin–Chitosan Nanocomposite for Extraction and Preconcentration of Multi-Class Emerging Contaminant Residues in Environmental Samples

Supplementary data

S1. Instrumentation

A Scientech ultrasonic cleaner (Labotec, Midrand, South Africa) with a volume of 5.7 L (internal dimensions: 300 × 153 × 150 mm) was used to facilitate the adsorption process. The ultrasonic system was equipped with a variable frequency and power setting. In this study, the frequency was fixed at 50 Hz and the emission power of 150 W. The analysis of the antibiotics was performed using an Agilent HPLC 1200 Infinity series, equipped with a photodiode array detector (Agilent Technologies, Waldron, Germany). Chromatograms were recorded at 290 nm. While an Agilent Zorbax Eclipse Plus C18 column (3.5 µm × 150 mm × 4.6 mm) (Agilent, Newport, CA, USA) was operated at an oven temperature of 25 °C. The mobile phase for separation of fluoroquinolones was composed of water (10 mmol L^{-1}) of phosphoric acid, the pH adjusted to 3.29 with triethylamine): acetonitrile (85.7:14.3, *v/v*) at a flow rate of 1.0 mL min^{-1}. All chromatographic experiments were carried out 25 ± 3 °C while the injection volume was 1.0 µL for all samples. For analysis of ß-blockers, CBZ and parabens, the mobile phase consisted of 30% water: 70% methanol mixture at a flowrate of 1.00 mL min^{-1}.

Table S1. CCD Matrix and analytical response: preconcentration of Beta-blockers and CBZ.

**Run**				**%Recovery**		
	pH	ET(min)	MA(mg)	ANTL	PPNL	CBZ
**1**	4.0	5.0	20.0	75.6	75.4	76.1
**2**	4.0	5.0	50.0	72.1	71.9	72.6
**3**	4.0	20.0	20.0	81.3	81.1	81.8
**4**	4.0	20.0	50.0	87.0	86.8	87.5
**5**	9.0	5.0	20.0	52.3	52.1	52.8
**6**	9.0	5.0	50.0	59.4	59.2	59.9
**7**	9.0	20.0	20.0	63.9	63.7	64.4
**8**	9.0	20.0	50.0	65.3	65.1	65.8
**9**	2.8	12.5	35.0	56.0	55.8	56.5
**10**	10.2	12.5	35.0	45.3	45.1	45.8
**11**	6.5	1.5	35.0	33.3	33.1	33.8
**12**	6.5	23.5	35.0	98.3	98.1	98.8
**13**	6.5	12.5	12.9	63.7	63.5	64.2
**14**	6.5	12.5	57.1	98.7	98.5	99.2
**15 (C)**	6.5	12.5	35.0	98.6	98.4	99.1
**16 (C)**	6.5	12.5	35.0	99.2	99.0	99.7
**17 (C)**	6.5	12.5	35.0	99.3	99.1	99.8
**18 (C)**	6.5	12.5	35.0	98.5	98.3	99.0
**19 (C)**	6.5	12.5	35.0	97.9	97.7	98.4

Table S2. CCD Matrix and analytical response: preconcentration of fluoquinolones.

**Run**				**%recovery**		
	pH	ET(min)	MA(mg)	DANO	ENRO	LEVO
**1**	4.0	5.0	20.0	60.5	65.5	63.9
**2**	4.0	5.0	50.0	64.0	67.4	65.0
**3**	4.0	20.0	20.0	78.9	78.9	81.7
**4**	4.0	20.0	50.0	84.4	84.4	85.2
**5**	9.0	5.0	20.0	74.9	78.1	80.2
**6**	9.0	5.0	50.0	75.2	85.2	86.1
**7**	9.0	20.0	20.0	80.2	89.7	91.2
**8**	9.0	20.0	50.0	81.1	90.1	87.2
**9**	2.8	12.5	35.0	54.5	54.5	56.8
**10**	10.2	12.5	35.0	21.0	24.8	23.0
**11**	6.5	1.5	35.0	55.4	55.4	52.7
**12**	6.5	23.5	35.0	98.1	98.1	99.7
**13**	6.5	12.5	12.9	47.1	47.1	43.1
**14**	6.5	12.5	57.1	98.1	101	99.5
**15 (C)**	6.5	12.5	35.0	99.9	99.9	96.7
**16 (C)**	6.5	12.5	35.0	99.8	99.8	97.1
**17 (C)**	6.5	12.5	35.0	98.9	102	98.1
**18 (C)**	6.5	12.5	35.0	100	100	96.7
**19 (C)**	6.5	12.5	35.0	98.1	98.1	96.9

Table S3. CCD Matrix and analytical response: preconcentration of parabens.

**Run**				**%Recovery**			
	pH	ET(min)	MA(mg)	MP	EP	PP	BP
**1**	4.0	5.0	20.0	73.0	75.0	73.5	73.8
**2**	4.0	5.0	50.0	79.0	81.2	79.5	79.8
**3**	4.0	20.0	20.0	89.0	92.3	89.5	89.8
**4**	4.0	20.0	50.0	88.0	87.4	88.5	88.8
**5**	9.0	5.0	20.0	56.0	54.6	56.5	56.8
**6**	9.0	5.0	50.0	63.0	64.8	63.5	63.8
**7**	9.0	20.0	20.0	71.0	75.4	71.5	71.8
**8**	9.0	20.0	50.0	75.3	73.3	75.8	76.1
**9**	2.8	12.5	35.0	60.0	59.8	60.5	60.8
**10**	10.2	12.5	35.0	56.0	54.7	56.5	56.8
**11**	6.5	1.5	35.0	21.3	24.7	21.8	22.1
**12**	6.5	23.5	35.0	96.7	97.6	97.2	97.5
**13**	6.5	12.5	12.9	69.0	71.2	69.5	69.8
**14**	6.5	12.5	57.1	99.7	98.7	100.2	100.5
**15 (C)**	6.5	12.5	35.0	98.7	97.8	99.2	99.5
**16 (C)**	6.5	12.5	35.0	97.6	97.6	98.1	98.4
**17 (C)**	6.5	12.5	35.0	98.5	98.1	99.0	99.3
**18 (C)**	6.5	12.5	35.0	98.3	98.4	98.8	99.1
**19 (C)**	6.5	12.5	35.0	97.9	98.3	98.4	98.7

Table S4. Analytical characteristics of DMSPE-HPLC-DAD method for determination of atenolol, propranolol and carbamazepine.

**Analytical performances**	**Atenolol**	**Propranolol**	**Carbamazepine**
**Linearity (μg L^{-1})**	LOQ-300	LOQ-400	LOQ-350
**Correlation coefficient (R^2)**	0.9987	0.9991	0.9989
**LOD (ng L^{-1})**	0.7	0.1	0.3
**LOQ (ng L^{-1})**	2.3	0.33	1.0
**Repeatability (%RSD)**	2.5	1.9	2.1
**Reproducibility(%RSD)**	4.3	3.4	3.1

Table S5. Analytical characteristics of DMSPE-HPLC-DAD method for determination of DANO, ENRO and LEVO.

Analytical performances	DANO	ENRO	LEVO
**Linearity (μg L^{-1})**	LOQ-950	LOQ-1000	LOQ-850
**Correlation coefficient (R^2)**	0.9987	0.9979	0.9990
**LOD (μg L^{-1})**	0.73	1.1	0.45
**LOQ (μg L^{-1})**	2.4	3.7	1.5
**Repeatability (%RSD)**	1.8	3.4	2.5
**Reproducibility(%RSD)**	3.0	2.8	4.4

Table S6. Analytical characteristics of DMSPE-HPLC-DAD method for determination of four parabens.

Analytical performances	Methyl -parabens	Ethyl--parabens	Propyl-parabens	Butyl-parabens
**Linearity (μg L^{-1})**	LOQ-250	LOQ-300	LOQ-250	LOQ-300
**Correlation coefficient (R^2)**	0.9987	0.9991	0.9989	0.9993
**LOD (ng L^{-1})**	0.8	0.2	0.5	0.4
**LOQ (ng L^{-1})**	2.7	0.67	1.7	1.3
**Repeatability (%RSD)**	2.7	1.5	2.1	2.3
**Reproducibility(%RSD)**	3.6	4.4	2.9	3.7

Table S7. Determination of atenolol, propranolol and carbamazepine in real water samples (ng L^{-1}, n = 3).

Samples	Analytes	Initial (ng L^{-1})	Found (ng L^{-1})	%R1	%RSD	Found (ng L^{-1})	%R2	%RSD
			50			100		
**Influent**	Atenolol	28.9±0.7	72.1±2.0	86.3	2.8	118±4	88.8	3.4
	Propranolol	20.6±0.5	66.3±3	91.3	4.5	110±3	89.7	2.7
	Carbamazepine	ND	47.2±1.6	94.4	3.3	93.3±1.5	93.3	1.6
**Effluent**	Atenolol	4.91±0.07	54.3±1.7	98.7	3.1	102±3	96.8	2.9
	Propranolol	7.65±0.05	55.4±1.9	95.4	3.4	105±5	97.1	4.9
	Carbamazepine	ND	48.4±2.1	96.8	4.3	95.5±1.6	95.5	1.7
**Tap water**	Atenolol	ND	49.4±1.4	98.8	2.8	99.2±2.0	99.2	2.0
	Propranolol	ND	48.9±1.7	97.7	3.5	98.9 ±3.0	98.0	3.0
	Carbamazepine	ND	49.6±1.3	99.1	2.6	99.5±4.1	99.5	4.0
**River water**	Atenolol	4.92±0.05	53.3±1.6	97.3	3.0	102±3	97.4	2.9
	Propranolol	2.05±0.03	50.6±2.1	97.0	4.2	99.1±1.9	97.0	1.7
	Carbamazepine	ND	49.5±1.1	98.9	2.2	99.1±2.3	99.1	2.3

Table S8. Determination of DANO. ENRO and LEVO in real water samples (ng L^{-1}, n = 3).

Samples	Analytes	Initial (ng L^{-1})	Found (ng L^{-1})	%R	%RSD	Found (ng L^{-1})	%R	%RSD
			5			20		
**Influent**	DANO	5.64±0.11	10.3±0.4	93.8	3.9	24.2±1.0	92.8	4.1
	ENRO	7.33±0.09	12.1±0.5	94.5	4.2	33.9±	100	4.3
	LEVO	ND	4.87±0.20	97.3	4.1	19.1±0.7	95.3	3.7
**Effluent**	DANO	1.65±0.03	6.52±0.21	97.3	3.2	21.3±0.66	98.1	3.1
	ENRO	2.07±0.07	6.88±0.17	96.2	2.4	21.0±0.6	94.4	2.9
	LEVO	ND	4.92±0.12	98.3	2.5	19.3±0.4	96.6	2.1
**Tap water**	DANO	ND	4.97±0.09	99.3	1.8	19.8±0.4	98.9	1.9
	ENRO	ND	4.98±0.07	99.6	1.5	19.8±0.4	99.0	1.8
	LEVO	ND	4.91	98.1	1.4	19.9±0.3	99.5	1.7
**River water**	DANO	2.40±0.03	7.14±0.16	94.8	2.3	21.5±0.3	95.6	1.4
	ENRO	3.12±0.05	8.02±0.2	97.9	2.5	22.4±0.3	96.3	1.3
	LEVO	ND	4.94±0.09	98.7	1.9	19.4±0.2	97.1	1.0

Table S9. Determination of MP, EP, PP and BP in real water samples (ng L^{-1}, n = 3).

Samples	Analytes	Initial	Found	%	%RSD	Found	%R	%RSD
			50			100		
**Influent**	MP	937±10	984±23	93.1	2.3	1078±32	94.3	3.0
	EP	1.37±0.11	46.2±1.2	89.7	2.5	92.7±3.2	91.3	3.5
	PP	781±11	828±25	94.3	3.1	924±24	96.1	2.6
	BP	ND	47.6±0.14	95.2	3.3	94.5±2.7	94.5	2.9
**Effluent**	MP	38.7±0.9	88.1±2.1	98.7	2.4	136±2.9	97.7	2.1
	EP	ND	48.7±1.0	97.3	2.1	96.5±1.8	96.5	1.9
	PP	43.1±0.8	92.7±1.8	99.1	1.9	141±2.5	97.9	1.8
	BP	ND	48.9±1.7	97.8	3.4	95.7±2.0	95.7	2.1
**Tap water**	MP	3.89±0.09	53.5±1.2	99.3	2.2	103±1.3	98.7	1.3
	EP	ND	49.0±1.2	97.9	2.4	99.1±1.5	99.1	1.5
	PP	4.81±0.05	53.8±1.3	98.0	2.4	104±1.7	99.3	1.6
	BP	ND	49.6±0.9	99.1	1.9	97.8±1.5	97.8	1.5
**River water**	MP	40.1±1.1	88.8±1.5	97.3	1.7	137±2.3	96.6	1.7
	EP	ND	48.5±0.9	96.9	1.9	97.1±1.8	97.1	1.9
	PP	10.7±0.8	59.7±1.4	98.3	2.4	108±1.4	97.0	1.3
	BP	ND	49.4±1.1	98.8	2.2	98.2±1.5	98.2	1.5

List of supplementary figures

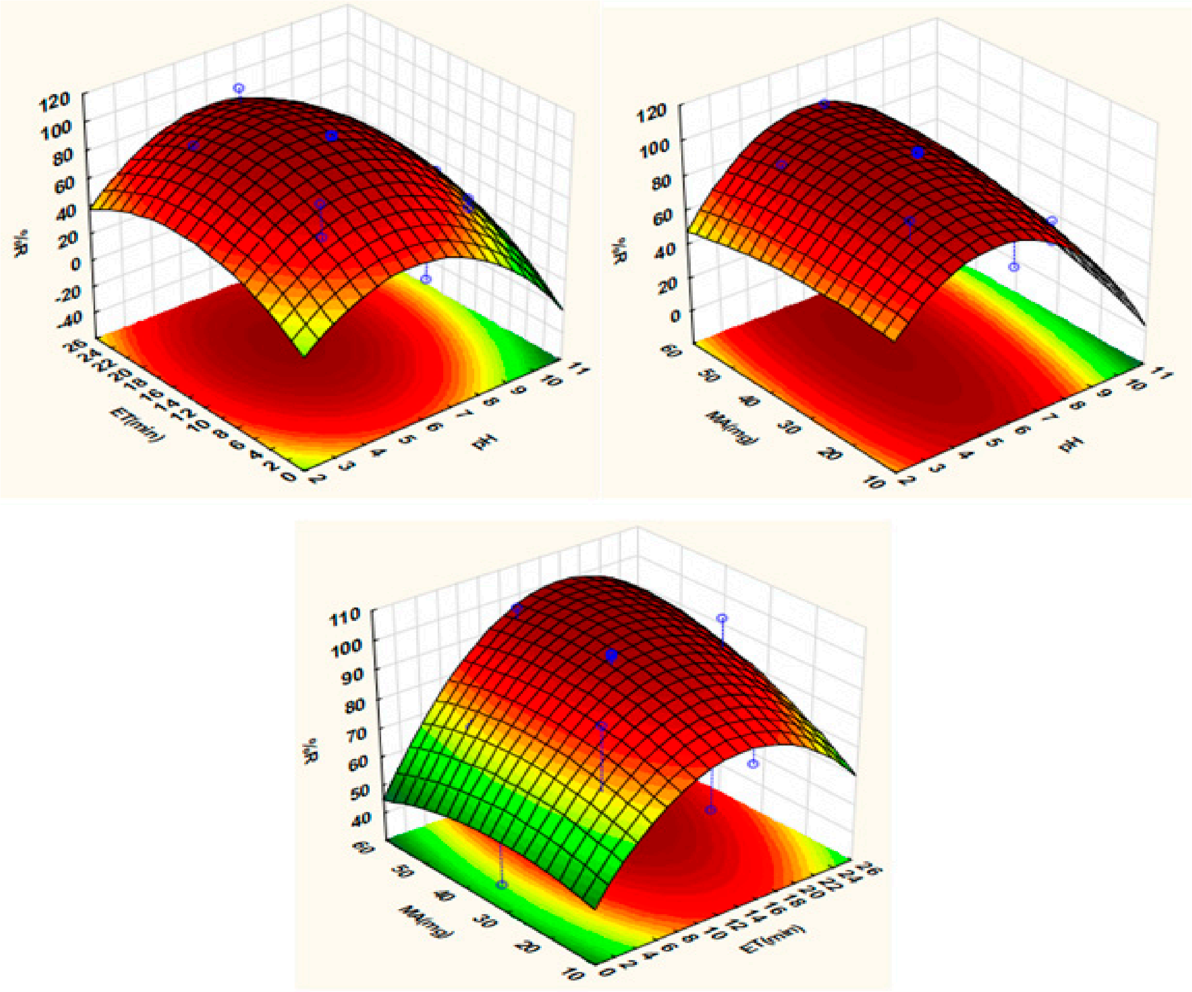

Figure S1. 3-D plots for the interaction of optimum parameters for preconcentration Beta-blockers and anticonvulsants.

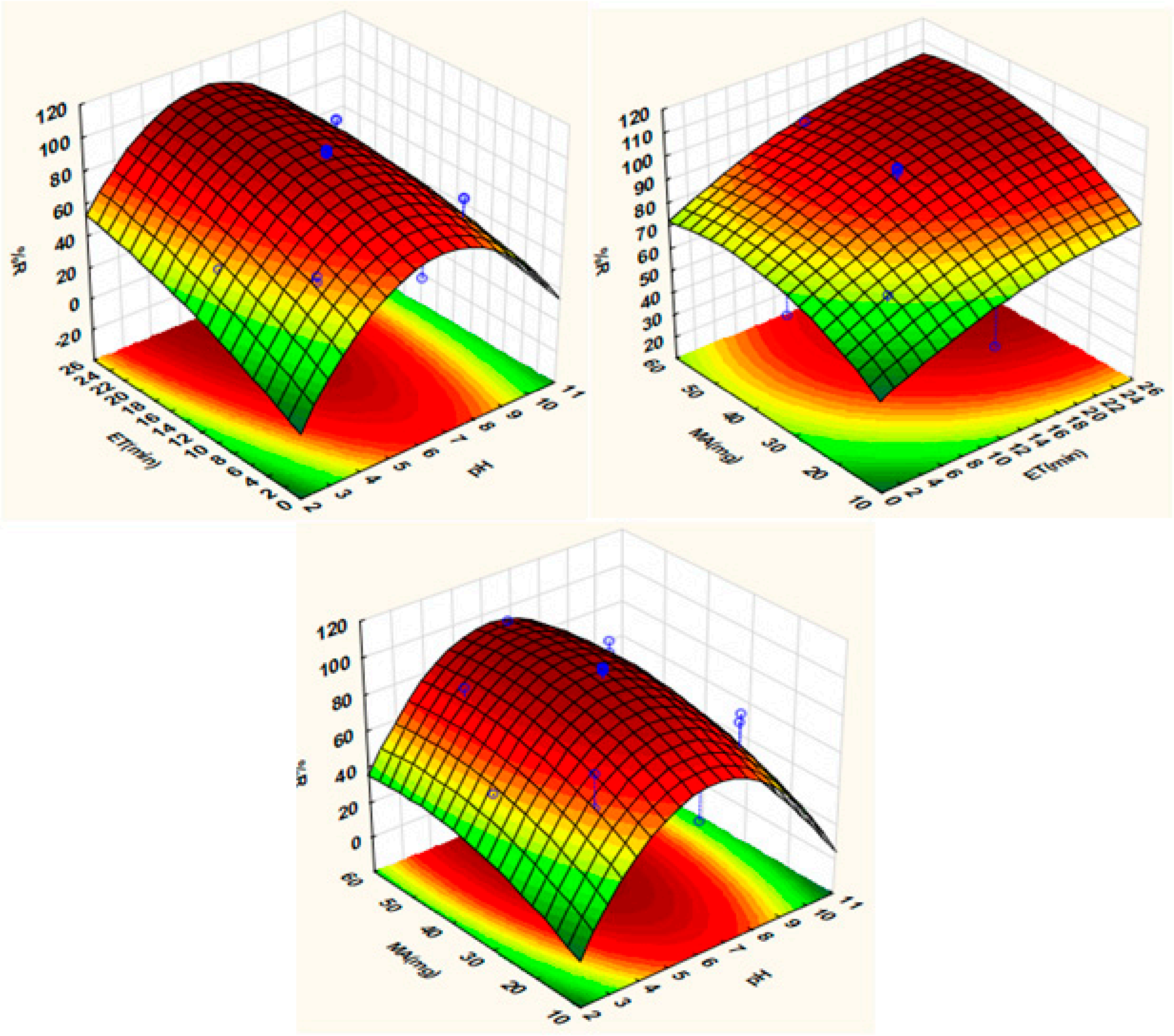

Figure S2 3-D plots for the interaction of optimum parameters for preconcentration of three fluoroquinolones.

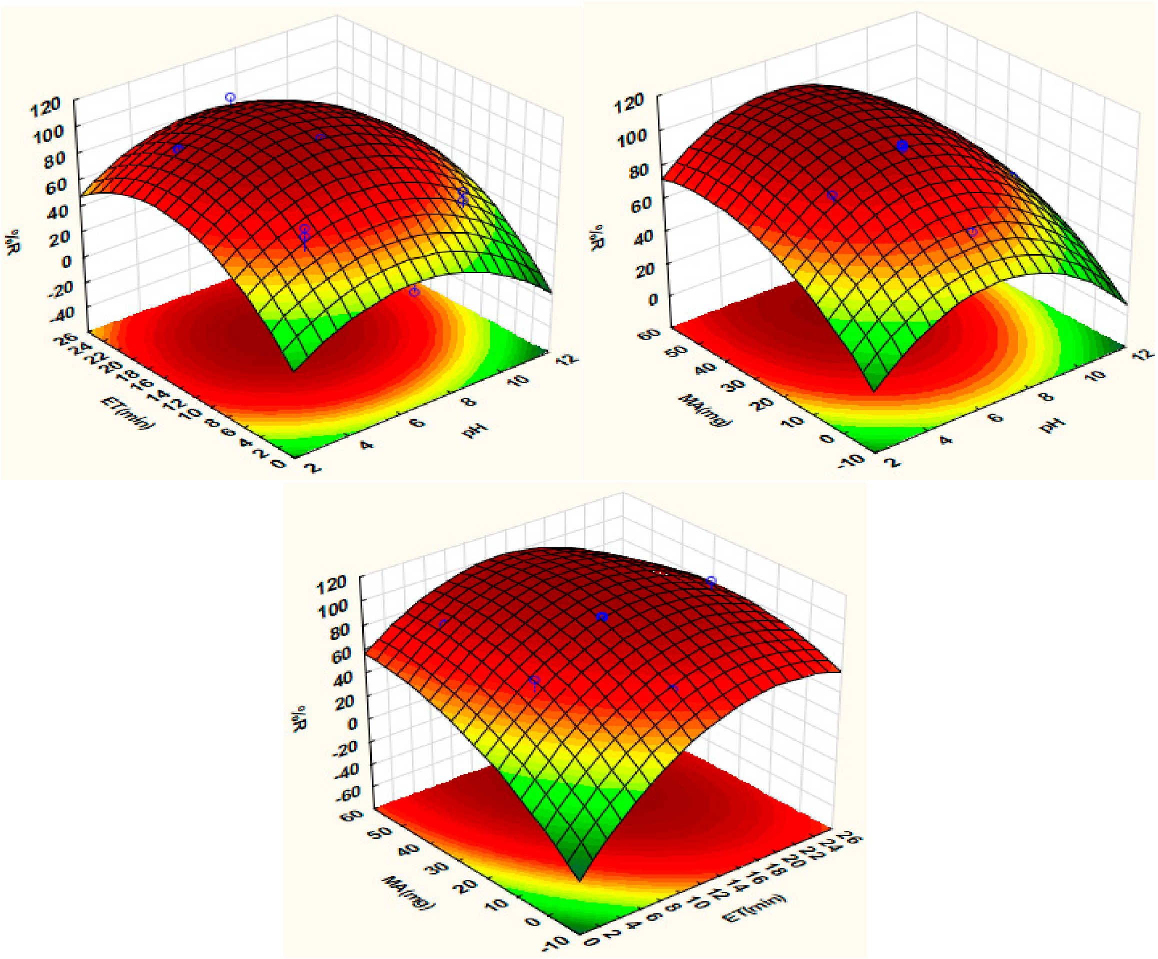

Figure S3. 3-D plots for the interaction of optimum parameters for pre-concentration of four parabens.

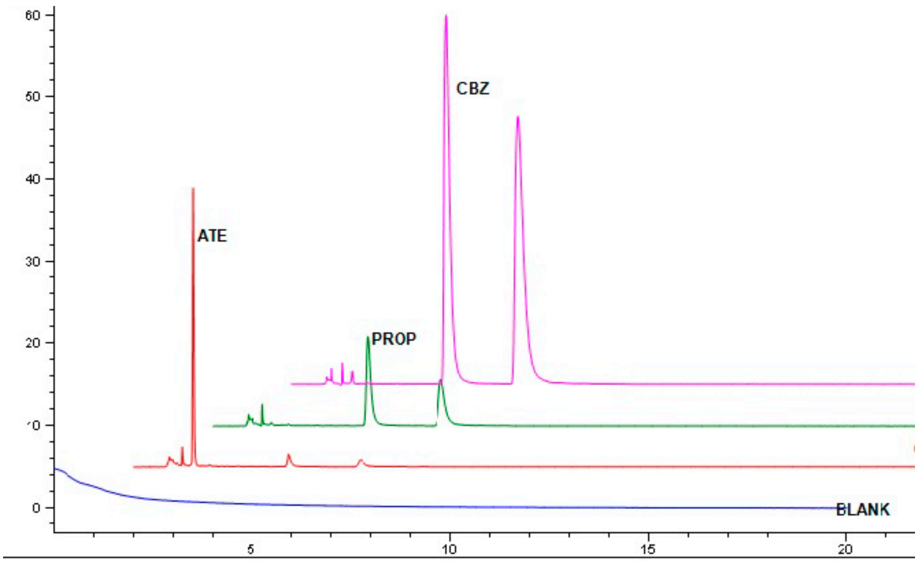

Figure S4. Typical chromatograms for preconcentration of Beta-blockers and anticonvulsants in influent samples.

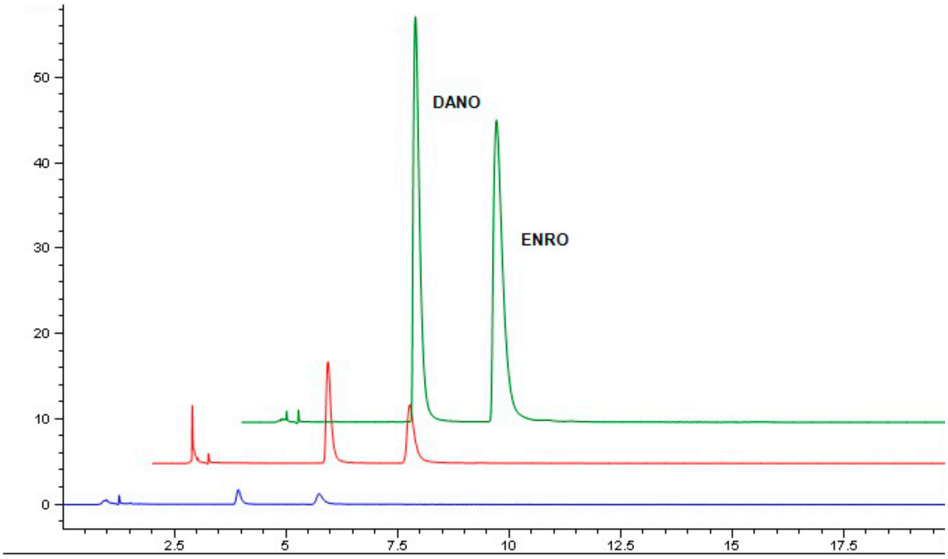

Figure S5. Typical chromatograms for preconcentration of fluoroquinolones in effluent samples.

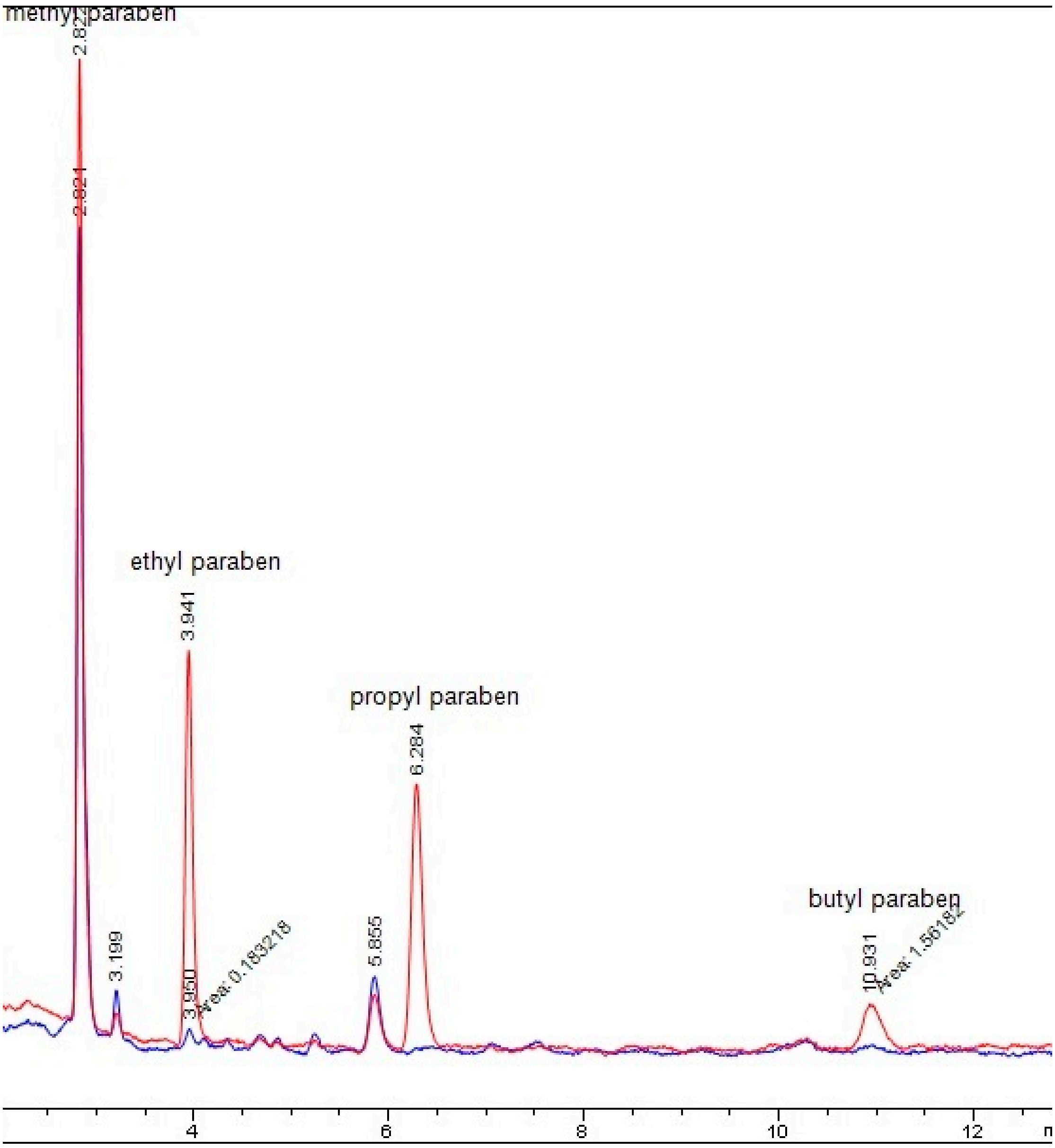

Figure S6. Typical chromatograms for preconcentration of parabens in influent samples.
